# Towards Standardization of Quantitative Retinal Vascular Parameters: Comparison of SIVA and VAMPIRE Measurements in the Lothian Birth Cohort 1936

**DOI:** 10.1167/tvst.7.2.12

**Published:** 2018-03-23

**Authors:** Sarah McGrory, Adele M. Taylor, Enrico Pellegrini, Lucia Ballerini, Mirna Kirin, Fergus N. Doubal, Joanna M. Wardlaw, Alex S. F. Doney, Baljean Dhillon, John M. Starr, Emanuele Trucco, Ian J. Deary, Thomas J. MacGillivray

**Affiliations:** 1 VAMPIRE project, Centre for Clinical Brain Sciences, University of Edinburgh, Edinburgh, UK; 2 Department of Psychology, University of Edinburgh, Edinburgh, UK; 3 Faculty of Medicine, University of Split, Split, Croatia; 4 UK Dementia Research Institute at the University of Edinburgh, Chancellor's Building, Edinburgh, UK; 5 Scottish Imaging Network, a Platform for Scientific Excellence (SINAPSE) Collaboration, Edinburgh, UK; 6 Centre for Cognitive Ageing and Cognitive Epidemiology, University of Edinburgh, Edinburgh, UK; 7 Division of Cardiovascular and Diabetes Medicine, Medical Research Institute, Ninewells Hospital and Medical School, Dundee, UK; 8 Alzheimer Scotland Dementia Research Centre, University of Edinburgh, Edinburgh, UK; 9 VAMPIRE project, Computing, School of Science and Engineering, University of Dundee, Dundee, UK

**Keywords:** retina, retinal microvasculature, retinal image analysis

## Abstract

**Purpose:**

Semiautomated software applications derive quantitative retinal vascular parameters from fundus camera images. However, the extent of agreement between measurements from different applications is unclear. We evaluate the agreement between retinal measures from two software applications, the Singapore “I” Vessel Assessment (SIVA) and the Vessel Assessment and Measurement Platform for Images of the Retina (VAMPIRE), and examine respective associations between retinal and systemic outcomes.

**Method:**

Fundus camera images from 665 Lothian Birth Cohort 1936 participants were analyzed with SIVA and VAMPIRE. Intraclass correlation coefficients (ICC) and Bland-Altman plots assessed agreement between retinal parameters: measurements of vessel width, fractal dimension, and tortuosity. Retinal–systemic variable associations were assessed with Pearson's correlation, and intersoftware correlation magnitude differences were examined with Williams's test.

**Results:**

ICC values indicated poor to limited agreement for all retinal parameters (0.159–0.410). Bland-Altman plots revealed proportional bias in the majority, and systematic bias in all measurements. SIVA and VAMPIRE measurements were associated most consistently with systemic variables relating to blood pressure (SIVA *r*'s from −0.122 to −0.183; VAMPIRE *r*'s from −0.078 to −0.177). Williams's tests indicated significant differences in the magnitude of association between retinal and systemic variables for 7 of 77 comparisons (*P* < 0.05).

**Conclusions:**

Agreement between two common software applications was poor. Further studies are required to determine whether associations with systemic variables are software-dependent.

**Translational Relevance:**

Standardization of the measurement of retinal vascular parameters is warranted to ensure that they are reliable and application-independent. This would be an important step towards realizing the potential of the retina as a source of imaging-derived biomarkers that are clinically useful.

## Introduction

Retinal microvascular features and their changes identified from the analysis of fundus camera images have been associated with cardiovascular disease, hypertension, stroke, dementia, and cognitive impairment.^1–5^ The development of semiautomated software applications, such as Singapore “I” Vessel Assessment (SIVA; National University of Singapore, Singapore), Vessel Assessment and Measurement Platform for Images of the Retina (VAMPIRE),^6–8^ Interactive Vessel Analysis (IVAN; University of Wisconsin, Madison, WI), Quantitative Analysis of Retinal Vessel Topology (QUARTZ),^9^ Retinal Analysis (RA; Department Ophthalmology & Visual Science, University of Wisconsin), and Automated Retinal Image Analyser (ARIA)^10^ has enabled the measurement of quantitative retinal parameters with increasing efficiency.^11^ While these measurements offer great potential to examine the role of microvascular pathology in the pathophysiology of cerebral and cardiovascular diseases,^12^ the applicability of retinal measurements in the clinical setting is yet to be fully established, partly due to methodologic limitations.

In the absence of ground truth measurement, the accuracy (i.e., the degree to which an instrument measures the true value of a variable) of such software applications cannot be determined. Method-comparison studies, however, assess how measurements from different software applications agree/differ. Agreement can be examined in two ways; firstly, by comparison of the absolute raw values produced by each software application (absolute agreement) and secondly, by comparison of the individual differences (i.e., differences between individual scores when ranked along a continuum) measured by each application (individual differences agreement). While some studies have identified several potential sources of variation, including physiologic characteristics of the eye, angle of imaging, image quality, cameras, thresholding and segmentation methods, and intraobserver variability,^13–15^ few studies have compared measurements from different software applications, which is the focus of this study. Current measurements of retinal vascular parameters might vary depending on the software from which they are derived. For instance, the absolute or “raw” value of the central retinal artery equivalent (CRAE; a summary of arteriolar vessel width) measured using software X might not be consistent with the absolute value of CRAE from the same image measured using software Y. A fundamental issue is whether differences in absolute (raw) measurements between the applications translate into meaningful differences in the detection of associations with systemic variables. If agreement across software in the measurement of individual differences is good (i.e., software applications X and Y rank individuals in a similar position along the measurement scale for a given retinal parameter), differences in absolute values might be less important. Systematic bias may not affect associations with systemic variables as long as the linear relationship between measurements from each method is the same. However, should retinal vascular measurements from fundus image analysis be applied as in optical coherence tomography measurements of the retinal nerve fiber layer in the diagnosis of glaucoma and other optical pathologies,^16^ or to determine inclusion in a clinical trial, a systematic bias would be very problematic.

Only moderate agreement of individual differences has been found previously between measurements of retinal vessel widths using IVAN and SIVA, meaning the error between the measurement and the true value was not constant for each software application. For instance, Yip et al. (*IOVS* 2012;53:ARVO E-Abstract 4113) reported an intraclass correlation coefficient (ICC) 0.516 and 95% confidence interval (CI) 0.41–0.61 for CRAE, and ICC 0.509 and 95% CI 0.40–0.6 for central retinal vein equivalent (CRVE). Significant intersoftware differences in CRAE and CRVE measurements (*P* < 0.001) from IVAN and SIVA also have been reported by Hao et al.^17^ SIVA returned systematically larger measurements of CRAE and CRVE compared to IVAN according to Wei et al.^18^ These findings indicate that agreement among software is not strong in terms of absolute measurements, and the low-to-moderate ICC values reported by Yip et al. (*IOVS* 2012;53:ARVO E-Abstract 4113) suggest that precision or agreement in the measurement of individual differences also may be poor.

Few studies have explicitly compared the strength of associations between retinal parameters and systemic variables across different applications. Yip et al.^19^ measured CRAE and CRVE using three software applications (RA, IVAN, and SIVA), and assessed the degree of agreement between their associations with systemic factors, including blood pressure, cholesterol levels, and body mass index. Though there were large differences in the absolute values of CRAE and CRVE among the software (CRAE, mean difference = −6.7 to −21.8 μm; CRVE −7.7 to −18.2 μm), Pearson's correlation coefficients (assessing individual differences in agreement for the retinal measures from each software) were high (*r*'s from 0.762–0.895) and there were no significant differences in the strength of their associations with systemic variables. Therefore, it remains unclear whether one software has greater predictive utility than another.

We used retinal imaging data from the Lothian Birth Cohort 1936 (LBC1936) to investigate the variation between two well-used software applications: SIVA and VAMPIRE. To the best of our knowledge, there has been no comprehensive assessment of the association between retinal parameters measured using different software applications beyond individual studies of summary vessel width measures and fractal dimension. We determined agreement in absolute (raw values) and individual (precision) differences between measurements of widths, tortuosity, and fractal dimension from SIVA and VAMPIRE. Furthermore, we determined the strength of association of measurements taken from both software applications with well-established systemic variables (blood pressure, inflammatory markers, and large-artery atheroma), and examined potential differences in the magnitude of these associations. We also discussed areas affecting reliability of semiautomated retinal measurements with a view towards standardization within the field.

## Methods

Ethical permission for the LBC1936 study was obtained from the Lothian Research Ethics Committee (Wave 1, LREC/2003/2/29), the Multi-Centre Research Ethics Committee for Scotland (Wave 1, MREC/01/0/56), and the Scotland A Research Ethics Committee (Wave 2, 07/MRE00/58). Written informed consent for participation in the study was obtained from all participants. The research was carried out in compliance with the Helsinki Declaration.

### Participants

Data were drawn from a subsample of the LBC1936 study. The LBC1936 comprises 1091 community-dwelling, healthy older adults, mostly free of diseases affecting the vasculature, and with a very narrow age range. Most participants completed the Moray House Test No. 12^20^ of verbal reasoning at a mean age of 11 as part of the Scottish Mental Survey of 1947 (SMS1947).^21,22^ Between 2004 and 2007, those residing in Edinburgh and the Lothians who may have taken part in the SMS1947, who then were approximately age 70, were contacted and invited to participate in the LBC1936 study. Recruitment and testing of this cohort has been described in detail previously.^23,24^ Data for the present study were obtained between 2008 and 2010 when the participants were approximately 73 years old (*N* = 866). Analyses were based on a subsample with retinal images suitable for analysis (*n* = 665).

### Retinal Image Analysis

Images of the right and left retinas were captured using a nonmydriatic camera and a 45° field of view (CRDGi; Canon USA, Inc., Lake Success, NY). Retinal parameters from one eye were measured using SIVA (version 3.0). If both images were of the same quality, the right eye was chosen (*n* = 343). If unavailable or ungradable, the image of the left eye was used (*n* = 322). Quality assessment was performed visually by a trained software operator following a standard protocol. The main reasons for image rejection included images being centered overly towards the macula (too few vessels visible); images with known pathologies, including asteroid hyalosis and cataract; and cases of very poor image quality, including out-of-focus images, eyelashes causing streaks across the photograph, small pupil size leading to dark or graining images, and overexposure. SIVA measurement and summarization methods have been described fully previously.^25,26^ Retinal parameters from the same images analyzed using SIVA were measured using VAMPIRE (version 3.1). VAMPIRE measurement and summarization procedures have been described in detail previously.^6,8,27–30^

A single trained operator was responsible for the visual assessment of automated measurements with each software application (MK, SIVA; SM, VAMPIRE), performing manual intervention where necessary, according to software-specific standardized measurement protocols. See Supplementary Table S1 for details of the main operator interactions. Supplementary Figure S1 presents the user interface of both software applications.

### Measures

#### Retinal Parameters.

CRAE, CRVE, arteriole-to-venule ratio (AVR), tortuosity (TORTa, TORTv), and fractal dimension (FDa, FDv) of the vasculature were calculated using SIVA and VAMPIRE. Separate arteriolar and venular measures were indicated by lowercase “a” or “v.” Measurements zones within which to measure retinal parameters were set (by SIVA and VAMPIRE) in relation to the center of the optic disc (OD) and its size. Vessel width measurements were derived from within Zone B (an annulus 0.5–1 disc diameter from the optic disc margin) and tortuosity and fractal dimension are measured from vessels within Zone C (an annulus 0.5–2.0 disc diameters from the disc margin). The labels of each retinal variable and the zone within which they are measured were the same for both software applications. The applications derived the same outcomes, but the underlying measurement algorithms differed. A description of all retinal parameters and zones is provided in Supplementary Table S2.

SIVA and VAMPIRE measured CRAE and CRVE in pixels from the images. The same method was applied for both software applications to convert pixel measurements to absolute measurements in microns. This was based on the assumption of an average OD diameter of 1800 μm in an adult human, adopted commonly in the literature.^31^ While with VAMPIRE the mean OD diameter (in pixels) of the entire sample was used to derive an image conversion factor (ICF), the procedure for SIVA was to calculate the ICF by measuring the OD diameter in a subsample of images (10%). We could have changed the conversion method of one software to match the other, thus eliminating one source of variability between software systems; however, the convention for conversion to micrometers was followed for VAMPIRE and SIVA according to their respective measurement protocols to better assess agreement without manipulation of the data to potentially increase agreement.

#### Systemic Variables.

Systemic variables were assessed, concurrently with obtaining the retinal images. We selected those factors that previously have been associated with retinal measures, including blood pressure, cardiovascular disease, diabetes, and inflammation,^32^ and those that have been used previously to examine and comprehensively adjust for vascular risk in the LBC1936.^33^ Those factors were: hypertension (self-reported history), mean systolic and mean diastolic blood pressure (mean of three sitting BP readings, mm Hg), ankle-brachial pressure index, carotid intima-media thickness, hemoglobin A1c, plasma high-density lipoprotein cholesterol, C-reactive protein, von Willebrand factor, and interleukin-6 (IL-6). All variables, with the exception of self-reported hypertension, were treated as continuous variables.

### Statistical Analysis

Analyses were conducted using SPSS V.21 (IBM, New York, NY) and R (version 1.0.136). All variables were examined for normality before analysis. Outlying values (±3 standard deviations [SD]) were winsorized to minimize the influence of extreme outliers without losing relevant data. Tortuosity, C-reactive protein, IL-6, and carotid measures were log-transformed to improve their distributions, which were positively skewed. Two-way mixed model ICCs were used to evaluate the extent of correspondence between two methods (SIVA and VAMPIRE) for measuring the same parameter (e.g., CRAE). The ICC quantifies this agreement, combining a measure of correlation with a test of the difference in means correcting for systematic bias and agreement based on chance alone. ICCs are thought to be more appropriate for assessing whether two methods for measuring a quantitative parameter provide similar results than Pearson's *r*, which measures the extent to which two variables are linearly dependent.^34^ Method-comparison studies have demonstrated that a perfect linear relationship does not necessarily reflect good or even moderate agreement as measured by ICC.^34,35^ In cases where a systematic bias in the data is observed, Pearson's correlation may indicate high correlation despite poor agreement between values as the linear relationship between measurements would be unaffected.^35^ ICC results were interpreted using the following criteria: 0.00–0.49 = poor, 0.50–0.74 = moderate, and 0.75–1.00 = excellent.^36^ Single measure coefficients and 95% CIs are reported.

We used Bland-Altman plots^37,38^ to provide a visual representation of how differences between the measurements relate to the mean across the full-range of values. The difference between two measurements (VAMPIRE–SIVA) was plotted against the average of the two measurements with the 95% limits of agreement (LOA), defined as the mean difference ± 1.96 × SD. Narrower 95% LOA indicates higher agreement. The presence of systematic, or fixed bias was assessed using a one-sample *t*-test comparing the mean difference and zero value. Proportional bias also was tested by determining whether the slope of the regression line significantly differed from zero.

Finally, we used bivariate correlations to assess the associations between retinal measurements and systemic variables. Where both variables were continuous, we used Pearson's correlation, and biserial correlation was used when one variable was dichotomous. To minimize the potential for type 1 errors, *P* values were adjusted according to the false discovery rate using the p.adjust() function in the statistical software R, using the method of Benjamini and Hochberg.^39^ Briefly, the false discovery rate method uses an ensemble of hypothesis tests and sets the *P* value, which results in 5% of those hypothesis tests being false-positives. We used Williams's test,^40^ implemented in R (paired.r command), to examine potential differences between SIVA and VAMPIRE in the magnitude of the correlations between each systemic variable and retinal measurements. Williams's test is used to test for significant differences in magnitude of correlations between a predictor variable (e.g., blood pressure) and competing criterion variables (VAMPIRE and SIVA measurements) that themselves are correlated.

## Results

**Figure 1:**
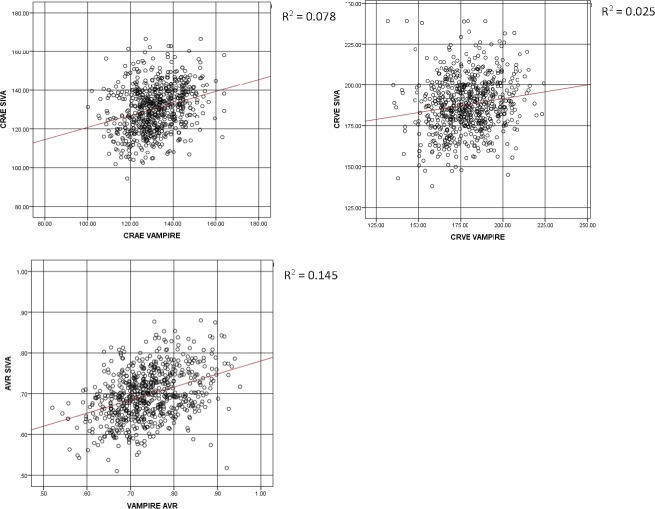
Vessel width. Scatterplots with regression lines of the relationship between vessel width (CRAE, CRVE, AVR) measurements from SIVA and VAMPIRE. CRAE and CRVE measured in micrometers. AVR, as a dimensionless measure, is not measured in standard units.

**Figure 2:**
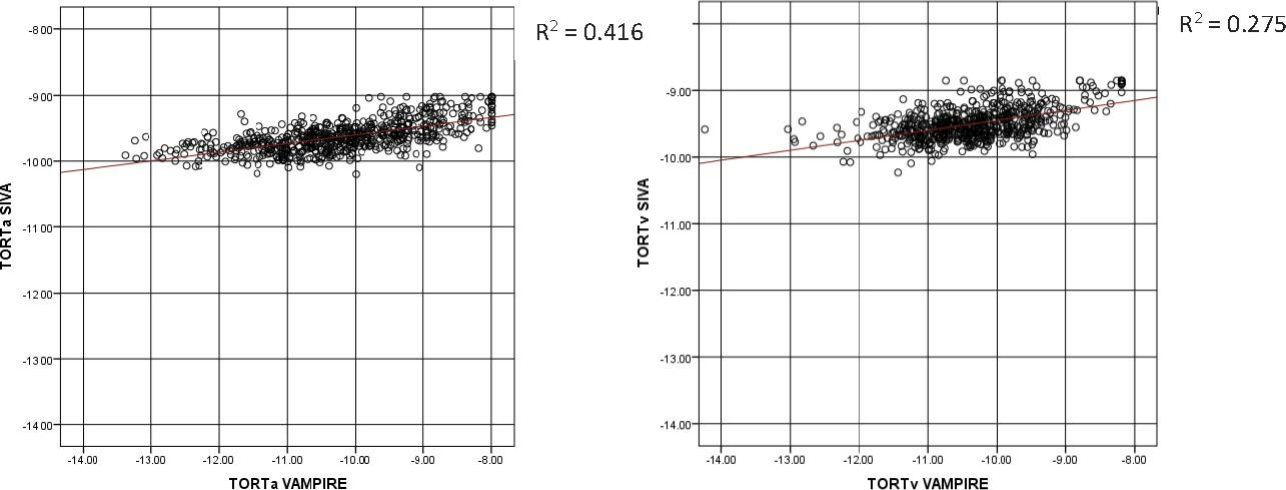
Tortuosity. Scatterplots with regression lines of the relationship between log-transformed tortuosity (TORTa, TORTv) measurements from SIVA and VAMPIRE. Tortuosity, as a dimensionless measure, is not measured in standard units.

**Figure 3:**
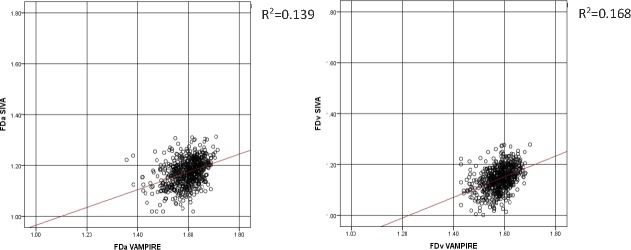
Fractal dimension. Scatterplots with regression lines of the relationship between fractal dimension (FDa, FDv) measurements from SIVA and VAMPIRE. Fractal dimension, as a dimensionless measure, is not measured in standard units.

Table 1 describes the characteristics of the study sample. Participants with at least one retinal measurement suitable for analysis, that is, the vasculature could clearly been seen in the image and was segmented by both software applications, thus enabling completion of the measurement process, (*n* = 665; 328 female; 337 male) had a mean age of 72.5 years (SD = 0.70) at the time retinal photographs were taken. Mean absolute values of retinal measurements from VAMPIRE and SIVA are reported in Table 2. Scatterplots of the relationship between retinal measurements using VAMPIRE and SIVA are presented for all variables (Figs. 1–3).

**Table 1:**
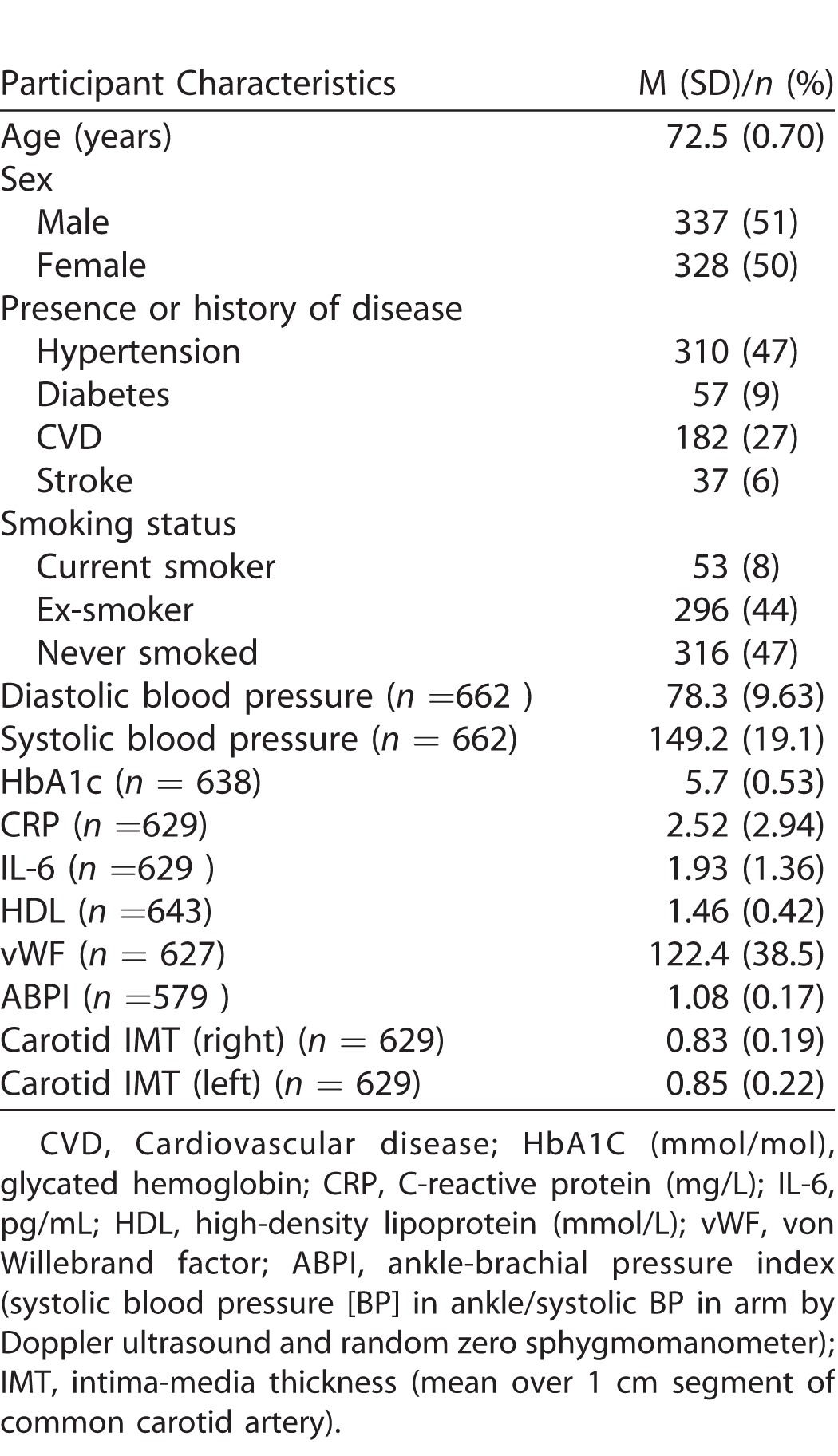
Characteristics of Participants With Images Analyzed by VAMPIRE and SIVA (n = 665; M (SD) for Continuous Variables and N (%) for Categorical Variables)

**Table 2:**
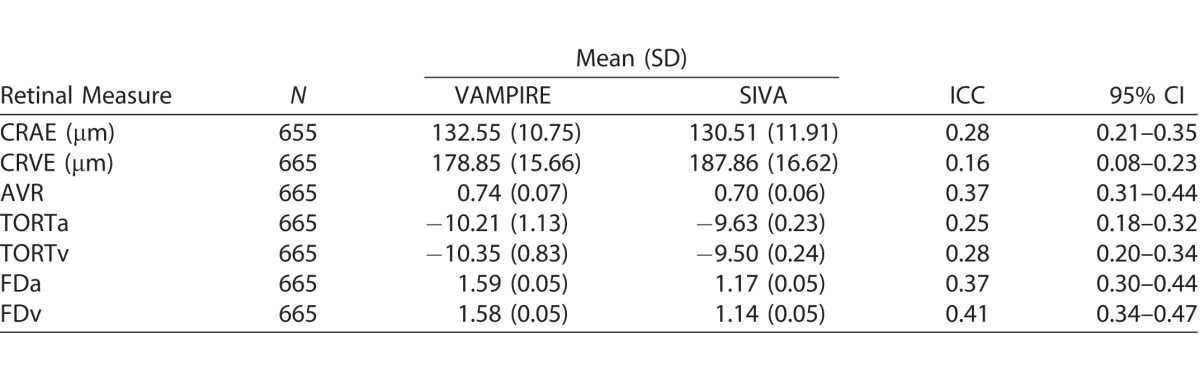
Intersoftware Variation in Retinal Vessel Measurements: ICCs

ICCs for correspondence of retinal parameter measures between SIVA and VAMPIRE imaging software measurements are shown in Table 2. ICCs indicated that agreement between all measures was poor. Agreement was poor between measures of vessel width (0.159–0.278) and tortuosity (0.254–0.276). Measures of fractal dimension and AVR demonstrated slightly better agreement (0.373–0.410).

**Figure 4:**
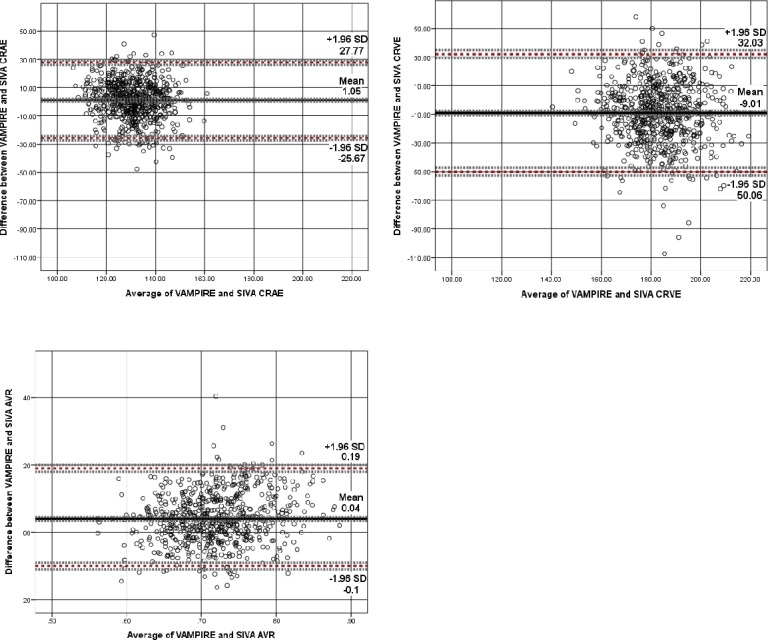
Vessel width. Bland–Altman plot of differences between CRAE, CRVE, and AVR measurements from VAMPIRE and SIVA, plotted against the average of the two methods. Broken red lines represent the mean difference (2 SD) of the difference (95% limits of agreement). Broken grey lines represent 95% CI on bias, upper, and lower limits of agreement. CRAE and CRVE measured in micrometers. AVR, as a dimensionless measure, is not measured in standard units.

**Figure 5:**
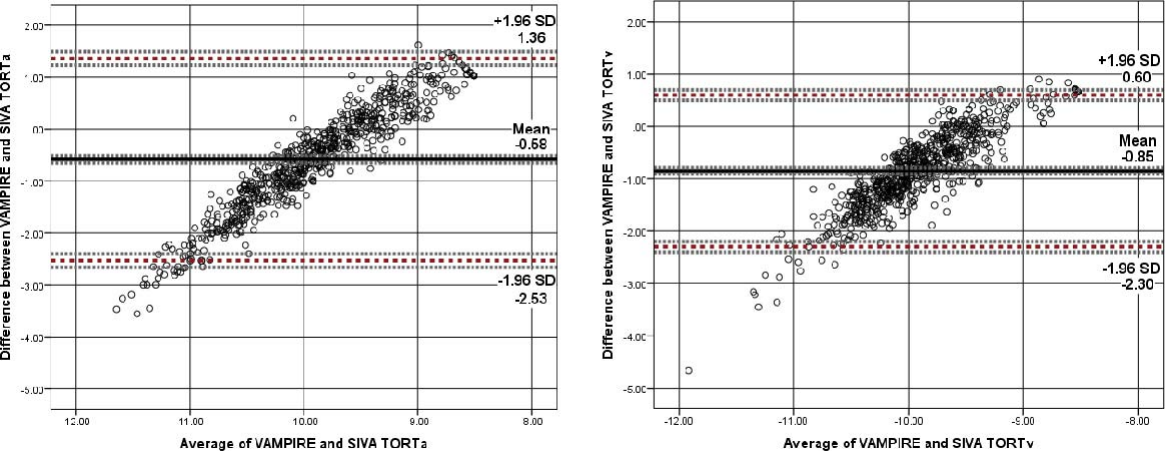
Tortuosity. Bland–Altman plot of differences between log TORTa and log TORTv measurements from VAMPIRE and SIVA, plotted against the average of the two methods. Broken red lines represent the mean difference (2 SD) of the difference (95% limits of agreement). Broken grey lines represent 95% CI on bias, upper, and lower limits of agreement. Tortuosity, as a dimensionless measure, is not measured in standard units.

**Figure 6:**
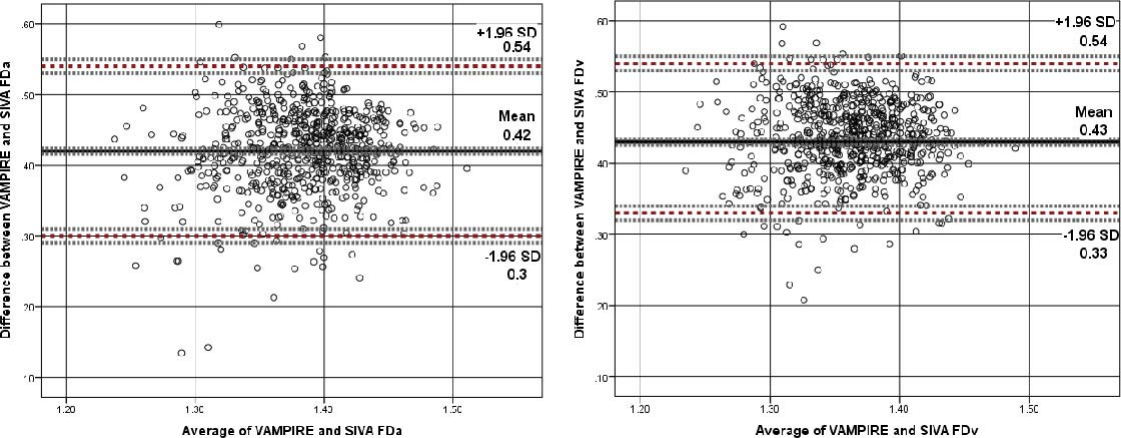
Fractal dimension. Bland–Altman plot of differences between FDa and FDv measurements from VAMPIRE and SIVA, plotted against the average of the two methods. Broken red lines represent the mean difference (2 SD) of the difference (95% limits of agreement). Broken grey lines represent 95% CI on bias, upper, and lower limits of agreement. Fractal dimension, as a dimensionless measure, is not measured in standard units.

Figures 4 to 6 show Bland-Altman plots illustrating the agreement between VAMPIRE and SIVA for measurement of seven retinal vascular parameters. As there is no “reference” standard, all differences were calculated such that SIVA measurements were subtracted from VAMPIRE. Average differences between VAMPIRE and SIVA, and systemic and proportional bias are described in Table 3.

**Table 3:**
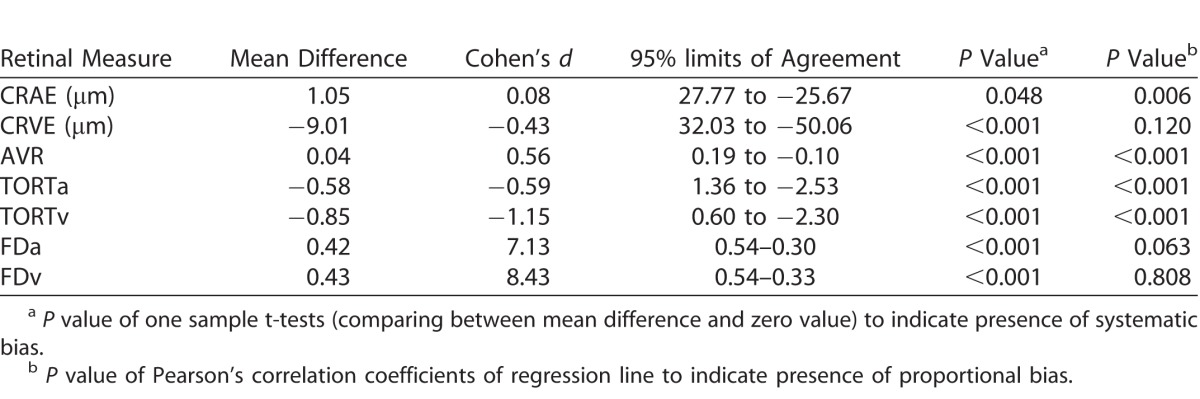
Agreement Analysis Between SIVA and VAMPIRE

The Bland-Altman plots indicated that systematic bias was present for all retinal measures. For CRAE (Fig. 4), VAMPIRE measures were on average 1.05 μm more than SIVA measures (95% LOA, 27.77 to −25.67, *d* = 0.08). CRVE demonstrated a greater average difference (−9.01 μm; 95% LOA, 32.03 to −50.06; *d* = −0.43) between VAMPIRE and SIVA, with SIVA measuring higher values on average than VAMPIRE. For AVR, the plots indicated moderate agreement (VAMPIRE measured 0.04 units more than SIVA), but there was significant proportional bias (*P* < 0.001).

In Figure 5 the plots demonstrated proportional bias in tortuosity (*P* < 0.001), with differences between VAMPIRE and SIVA at different magnitudes of tortuosity. For low tortuosity values, VAMPIRE measured considerably lower values than SIVA, whereas for higher tortuosity values VAMPIRE measured higher values than SIVA, indicating that generally VAMPIRE records a greater dynamic range for tortuosity values. Overall the mean difference in TORTa was −0.58 (95% LOA, 1.36 to −2.53, *d* = −0.59). The mean difference in TORTv was −0.85 (95% LOA, 0.60 to −2.30, *d* = −1.15).

Mean difference in FDa (see Fig. 6) was 0.42 (95% LOA, 0.54–0.30, *d* = 7.13) with plots indicating proportional bias such that there was better agreement for higher values of FDa and greater degree of variability for low mean FDa values. Higher values of FDv also demonstrated better agreement than lower FDv values. Mean difference was 0.43 (95% LOA, 0.54–0.33, *d* = 8.43).

Tables 4 and 5 show the associations between systemic measures and retinal parameters measured by SIVA and VAMPIRE. While most significant associations between retinal parameters and systemic variables were found for only one or other software package, there were six instances of consistent significant associations for measurements from SIVA and VAMPIRE. There were significant negative associations between retinal vascular measures from both software and mean systolic and diastolic BP: diastolic BP (CRAE, *r*'s from −0.183 to −0.118; AVR, *r*'s −0.162 to −0.148; FDa, *r*'s −0.169 to −0.107) and systolic BP (CRAE, *r*'s −0.177 to −0.112; FDa, *r*'s −0.127 to −0.118). These associations remained significant after correcting for false discovery rate. There was one significant positive association between IL-6 and TORTv for SIVA (*r* = 0.098) and VAMPIRE (*r* = 0.096). These associations did not survive correction for false discovery rate. Supplementary Figure S2, as an example of one such instance of significant and consistent associations across software packages, shows a visual representation of the similar magnitudes of association of measurements from SIVA and VAMPIRE with diastolic BP. All other significant associations were found for only one or other software platform.

**Table 4:**
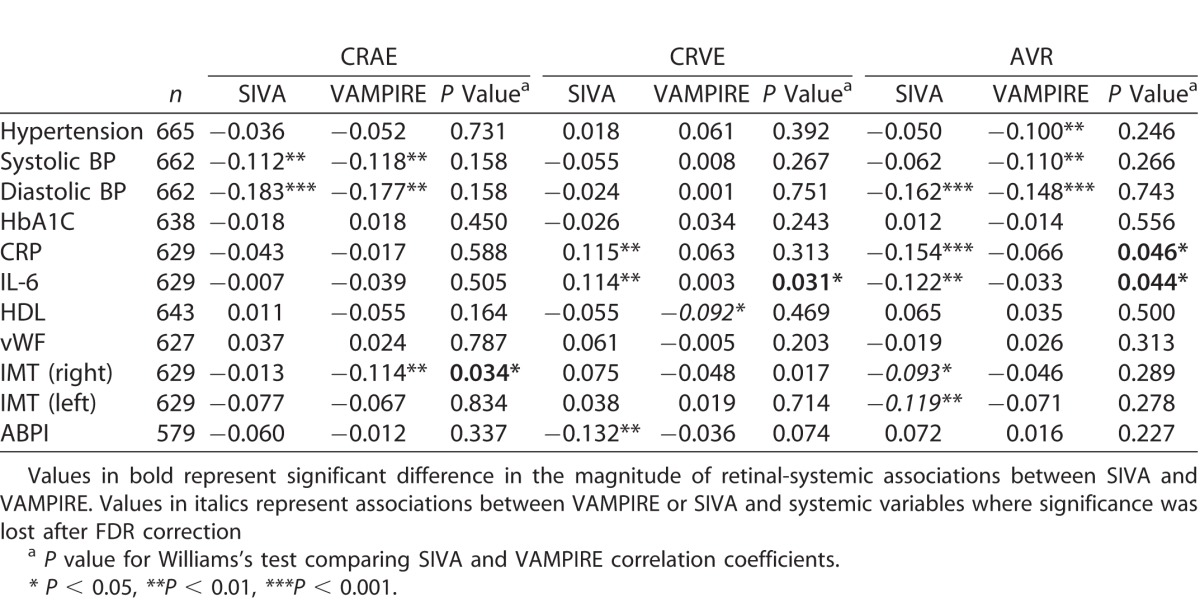
Association of Retinal Vessel Width Measurements From VAMPIRE and SIVA With Systemic Variables

**Table 5:**
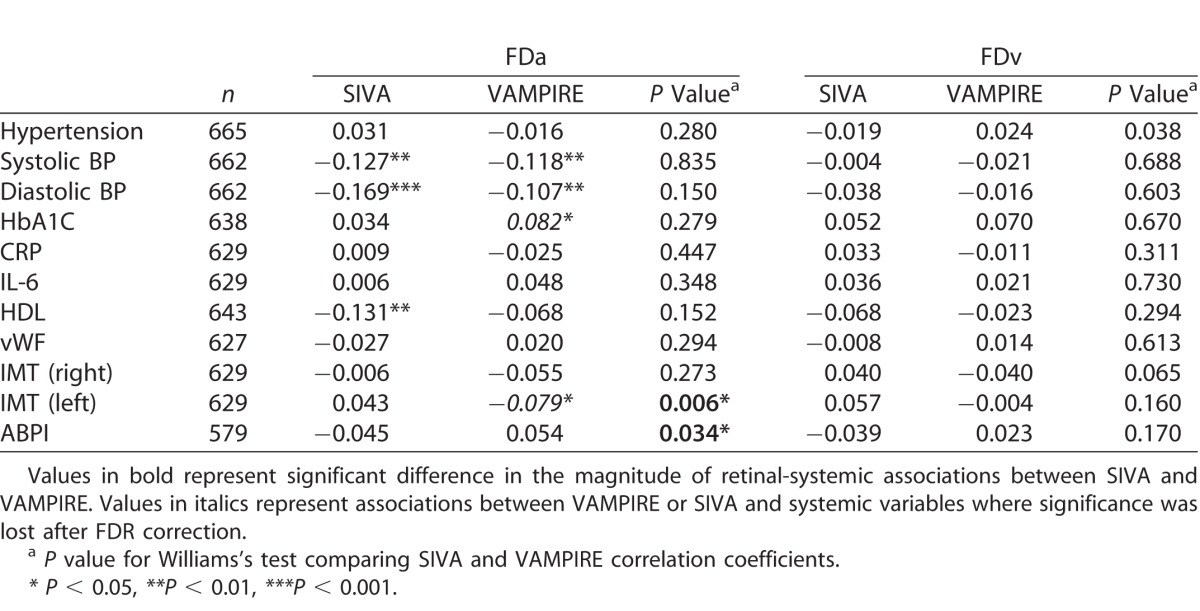
Association of Retinal Fractal Dimension and Tortuosity Measurements From VAMPIRE and SIVA With Systemic Variables

**Table 5:**
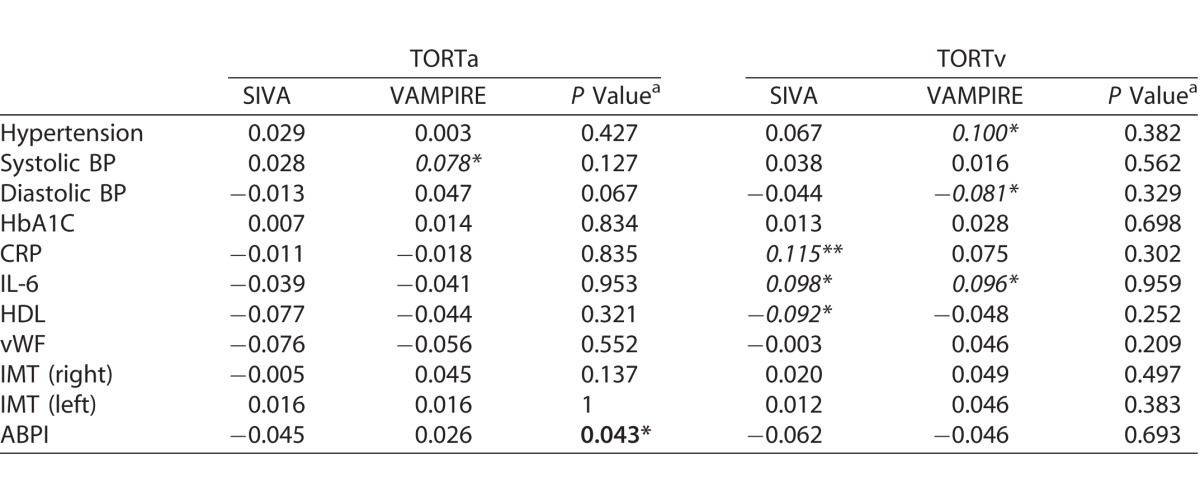
Extended

Williams's test indicated significant differences in the magnitude of retinal-systemic associations between SIVA and VAMPIRE in 7 of 77 comparisons. Four demonstrated a significantly stronger association with SIVA measurements than VAMPIRE, these were CRVE and IL-6 (*r* = 0.114 vs. *r* = −0.003; *P* = 0.03), AVR and C-reactive protein (*r* = −0.154 vs. *r* = −0.066, *P* = 0.05), AVR and IL-6 (*r* = −0.122 vs. *r* = −0.033, *P* = 0.04), and TORTa and ankle-brachial pressure index (*r* = −0.045 vs. *r* = −0.026, *P* = 0.04). The three associations where VAMPIRE demonstrated significantly stronger associations with systemic variables than SIVA were CRAE and right carotid intima-media thickness (*r* = −0.114 vs. *r* = −0.013, *P* = 0.03), FDa and left carotid intima-media thickness (*r* = −0.079 vs. *r* = 0.043, *P* = 0.01), and FDa and ankle-brachial pressure index (*r* = 0.054 vs. *r* = −0.045, *P* = 0.03). It should be noted that in two of these cases significant differences in magnitude were based on comparison of two small and nonsignificant correlations lying on either side of zero (FDa and ankle-brachial pressure index *r* = −0.045 vs. *r* = 0.054; TORTa and ankle-brachial pressure index *r* = −0.045 vs. *r* = 0.026).

Overall, the association between SIVA and VAMPIRE retinal vessel width, fractal dimensions and tortuosity measures was poor, and assessment of agreement between measures using Bland-Altman plots indicated the presence of systemic and proportional bias for the majority of parameters. Of the 77 retinal-systemic associations examined, six were significant for SIVA and VAMPIRE measurements. Five of these remained significant following correction for false discovery rate. Significant differences between software in the strength of correlation with systemic variables was found for seven of 77 comparisons.

## Discussion

Examination of Bland-Altman plots indicated that systematic bias was present for all retinal measurements; depending on the specific parameter, one software reported consistently higher or lower measurements than the other. Proportional bias, indicating variability in differences across the measurement range, also significantly contributed to a lack of agreement for most retinal parameters. Therefore, our findings did not support equivalence of retinal vascular measurements between software applications indicating that absolute measurements were not interchangeable. An important consequence was that values from different systems would require standardization for data pooling or meta-analysis. Individual differences agreement as measured by ICC also were poor, with all being considerably lower than the proposed 0.90 minimum level of agreement for measures to be used interchangeably in clinical practice.^41^ This leaves uncertainty over whether associations with systemic variables also are software dependent. Williams's tests showed significant differences in correlation magnitude for seven of 77 comparisons; however, conclusions regarding the equivalence of SIVA and VAMPIRE associations with systemic variables should be drawn cautiously given the low number of significant retinal-systemic associations after false discovery rate adjustment from either software found in the current sample. It is important to note, however, that the weak associations reported here do not imply poor retinal parameters. Our relatively homogeneous sample may have restricted the range of values required to identify stronger correlations. Associations might be stronger in clinically relevant samples. Comparability of retinal-systemic variable associations in broader populations, including those with disease, should be examined. Nevertheless, our results remain of value as an examination of agreement in a well-characterized sample of healthy older adults.

Current results correspond with, though ICCs still are lower than, those of Yip et al. (*IOVS* 2012; 53:ARVO E-Abstract 4113), who reported moderate agreement between the width measurements obtained from SIVA and IVAN in a middle-aged population. Associations between SIVA and VAMPIRE and systemic parameters were weaker than those found by Yip et al.^19^ with SIVA, IVAN, and RA. Sample age differences may account for the weaker results in our study as stronger cross-sectional associations have been reported in middle-aged compared to older populations.^42^ Few significant retinal-systemic variable associations were found for either SIVA or VAMPIRE measures in our study, possibly due to the sample being relatively disease-free with little variation in some parameters of interest. There currently are no clinically defined nor largely agreed cutoffs indicating the degree to which intersoftware retinal measurement differences are acceptable. For equivalence to be resolved in future studies, it will be necessary to determine the boundaries within which it is acceptable for absolute values to differ between methods without obscuring true morphologic differences. If retinal parameters are to be used as biomarkers and diagnostic indicators, it will be necessary to ensure that retinal measurement values reflect the state of the vascular system, be that healthy or diseased, and are not an artefact of the software being used.

At the current stage of development there are uncertainties regarding the reliability (accuracy) of measurements from both software applications. As we cannot directly measure retinal vessels in vivo there is no ultimate ground truth upon which to assess measurements from fundus camera image analysis. “Errors” cannot be defined as in metrology or physics, as our references themselves are affected by uncertainty. Such uncertainties are likely to contribute to the lack of agreement in the current results.

Lack of agreement can arguably be traced to two classes of factors. First, error variance may be introduced by factors that affect both software applications (See Supplementary Discussion). Second, there may be factors specific to either SIVA or VAMPIRE, such that one has good reliability while the other does not. In the following section we focus on the latter, and the impact these issues may have had on the results reported. Each factor provides an opportunity for progressing towards greater standardization of retinal measurements from fundus camera images.

The low agreement of some measurements may result from different solutions to obtaining retinal parameters (i.e., the combination of image postprocessing techniques and numerical algorithms) implemented in SIVA and VAMPIRE. This leads to the possibility that some measurements from one application may have better reliability than those from the other, which again would result in poor intersoftware agreement. The extent to which such differences are relevant for clinical investigations must be determined. The following examines briefly the eight main procedures involved.

### 

#### 

##### Vasculature detection

Retinal vessel measurements depend crucially on the detection of the retinal vasculature. Simply put, this involves the generation of a map showing which pixels are classified as either vessel or background. However, the maps generated by different algorithms for the same images will vary in terms of which vessels are detected (especially thin ones), and of their width and centerlines, for example. VAMPIRE uses a modified 2-D Gabor wavelet-supervised classification algorithm for automatic vessel detection^43^ and locates centerlines through skeletonization of the binary vessel map.^44^ SIVA incorporates Daubechies wavelet, trench, and curvature-based segmentation to identify and extract retinal vascular structure with a modified trench detection algorithm applied in vessel segmentation to locate the centerlines of the vessels.^26^ Unfortunately, as segmentation data were not available from both software packages, we could not assess the effect of these differences explicitly. Finally, threshold levels for the identification of very small vessels may differ.^45^

##### Determination of OD diameter

Differences in the determination of the OD diameter, through automatic software detection or inter/intraoperator differences also may influence measurements as the regions in which vessels are measured are defined by OD diameter. For example, in the case of AVR, the average vessel width will change according the size of the radius of Zone B and the inclusion and exclusion of vessels in the AVR calculation also will differ according to the region set by the OD diameter. OD measurements were not available from both software tools, precluding the assessment of agreement and effect of different OD diameter determination.

##### Vessel Width

Accurate quantification of vessel widths using semiautomated computer software has proven difficult.^46^ The lack of an absolute, objective definition of the retinal vessel boundary^8,7^ and of ground truth in vivo increases the uncertainty of measurements, normally validated against experts' annotations obtained from interactive packages.^46,47^ Computer-assisted quantification of vessel width from fundus camera imaging measures the width of the reflective erythrocyte column. As the surrounding clear plasma zone is not measured, true vessel diameter is underestimated.^48^ The vessel edge is not clear due to loss of intensity with reducing column depth at the boundary, which makes it difficult to determine whether individual pixels at the vessel edge belong to a vessel. While this is not likely to cause a large degree of variation, differences found in average single vessel width from each quadrant across 20 images measured using ARIA and IVAN (5.56–7.94 and 7.44–19.73 μm for arteriolar and venular width, respectively) were explained due to the use of different methods in defining the vessel edge by the two systems (Silvestri V, et al. *IOVS* 2012; 53:ARVO E-Abstract 2178).

##### Arteriole-to-Venule Ratio

Although one of the most standardized retinal measurements, AVR from different applications will differ as it is based on CRAE and CRVE, which vary depending on factors, such as the number of vessels measured. In our study, differences between AVR from VAMPIRE and SIVA ranged from 0.16 to 0.40. Based on average CRAE and CRVE values from SIVA (130.51–187.86 μm) and VAMPIRE (131.55–178.85 μm), a higher average AVR is found using VAMPIRE (0.74 vs. 0.70) due to the smaller difference between average artery and vein widths.

##### Tortuosity

Differences in the vessel segments that SIVA and VAMPIRE sample for tortuosity and in how these are combined into a single measure per image (i.e., the use of mean, weighted mean, or median to derive a single tortuosity value) are likely. Poor agreement of tortuosity values also could be due to validation differences (testing against clinical judgement after conversion to a 3- or 4-point scale, testing against different ground truth sets, annotated in turn by different experts), and the use of different computational definitions of tortuosity measured within different numerical ranges.^29^

##### Fractal Dimension

FD measurements are sensitive to variations in segmentation, image acquisition angle, and FD calculation algorithms.^15^ Differences between software in vessel detection and in the fractal computation in our study will have contributed to the different values that were returned. SIVA performs monofractal analysis using the box-counting method,^45^ whereas VAMPIRE performs monofractal using box counting and multifractal analysis using the generalized sand-box method.^49^ It should be noted, however, that in our experiments the multifractal VAMPIRE FD used in the current analysis had better agreement with FD analyzed by SIVA (ICCs from 0.373 to 0.410) than a monofractal FD calculated by VAMPIRE (ICCs from 0.192–0.195).

##### Algorithm Based Measurement

The current practice of estimating vessel width relies upon derived parameters (CRAE and CRVE) that are based on an algorithm, in itself is an estimation. As vessel width is not derived from direct measurement of a physiologic or anatomic feature, the reliability of the measurement, thus, is dependent on reliability of the algorithm. Algorithms also are used in measurement of other parameters that have proven difficult to quantify directly, such as tortuosity, for which numerous algorithm variations exist attempting to capture the curvature of vessels.

##### Pixel to Micron Conversion

For the purposes of comparison across imaging systems and software packages, pixel measurements of CRAE and CRVE frequently are converted to standard units of length (i.e., micrometers). The same approach was used in this study for SIVA and VAMPIRE (1800 μm/average OD diameter), as recommended by the SIVA protocol.^26^ However, assuming the make and model of the camera system and the angle of acquisition are consistent, then the conversion factor depends on the average OD diameter measurement in the specific patient sample. Considerable variation exists in OD size within and between populations.^50^ The use of a sample-specific conversion factor based on the sample average OD diameter (and on assumed 1800 μm average OD diameter) may have important implications for retinal measurements; this becomes evident when retinal measurements in micrometers based on the standard image conversion factor (ICF = 1800 μm/average OD diameter) are compared to those calculated using a conversion factor unique to an individual (1800 μm/individual OD diameter). We ran this test with a set of 10 LBC1936 images (two images selected at each quintile point of OD diameter range), converting VAMPIRE CRAE and CRVE measurements from pixels to micrometers using the standard and an individual conversion factors (See Supplementary Table S3). Individual OD diameters varied from the sample mean by up to 367 pixels (the average diameter being 428.92 pixels, SD = 48.14); this resulted in conversion factor differences of up to 3.6 (sample data ICF range = 6.35–2.76 vs. standard whole sample ICF 4.28). Subsequent CRAE and CRVE from the same image differed by up to 58 and 76 μm, respectively (the averages of standard and individual ICF measures of CRAE and CRVE being, respectively, 134.57 vs. 132.32 and 182.07 vs. 183.17) and the difference between standard and individual conversion factor produced CRAE and CRVE mean measurements was significant (*P* < 0.05; see Supplementary Table S4).

Crucially, the variation introduced by using a standardized conversion factor (up to 76 μm) is larger than differences reported between patient groups (e.g., CRVE of 218 μm in lacunar stroke vs. 208 μm in cortical stroke),^5^ and consequently may mask true differences between individuals. Using pixel measurements in statistical analyses, other factors being equal, removes the uncertainty introduced by pixels-micrometers conversion factors. Measurements in micrometers always can be obtained for the ophthalmologist's benefit, but must be interpreted carefully within the limits of the approximations or standardizations implied by the conversion method.

Some factors introducing uncertainties in retinal imaging are common to other areas of medical imaging.^51–53^ For example, speckle tracking echocardiography measurements were dependent on the algorithm applied by the specific software system used.^51,53^ Different algorithms also were a contributing factor to the poor agreement between three widely used software applications with myocardial perfusion imaging.^52^ A model eye allowing calibration with measurements of a known size could help to improve accurary.^54^ Phantoms of a known-size have been used in computed tomography, magnetic resonance imaging, and positron emission tomography imaging allowing for accuracy and variance to be analyed.^55,56^ Phantoms of the eye, including the retinal vasculature with simulated blood flow and known dimensions, could enable the calibration of measurements.

Our study has a number of strengths. We expanded upon the current literature in two ways, first by assessing agreement between measurements from two software applications on a comprehensive range of retinal vascular parameters. Previous studies have focused on individual parameters, for example, vessel width or fractal dimension and differences between software applications (Yip W, et al. *IOVS* 2012;53:ARVO E-Abstract 4113; Silvestri V, et al. *IOVS* 2012;53:ARVO E-Abstract 2178).^15,18^ Our study extended analysis to measures of tortuosity and we examined all parameters within a single dataset. Second, we assessed the equivalence of a range of retinal parameter associations with systemic variables across software applications, which also has been limited previously to vessel width measurements. The relatively large sample of healthy older adults was taken from a birth cohort with a very restricted age range, with similar geographic background, and mostly free of illnesses known to affect the vasculature, which enabled us to examine differences between the software applications without the risk of confounding by these important variables. The use of one trained operator for all image analyses in each of the software applications reduces potential for error due to interoperator variability.

Limitations of the study also should be noted. To the best of our knowledge, no extensive quantitative study of the stability of retinal measurements as a function of image quality (e.g., resolution, acquisition protocol, instruments) exists to date. At the current stage of software development, validation is not collaborative across research groups. Therefore, there are known differences between software in the formulas and methodology of retinal measurements which are beyond the scope of discussion in the current study. The absence of OD detection and segmentation data from both software applications leaves the agreement of and effect of these differences to be explored explicitly in further studies. Our study was limited to a comparison of two commonly used software packages. Future studies should assess agreement of measurements from a wider range of available software packages. A comprehensive analysis of agreement, performed by an international consortium, would be of great value in further clarifying the current standards of agreement and in extending the discussion towards greater standardization across software applications.

## Conclusion

Semiautomated retinal vasculature analysis measurements seem to be software-dependent. Based on the current results, we recommend caution when making inferences regarding the equivalence of associations between retinal measures from SIVA and VAMPIRE (and other similar software applications) and systemic variables, due to the limited number of corresponding associations between retinal measures and systemic variables found. It also is important to consider the variability in measurements when comparing results of retinal vascular measurements from different studies as some factors that contribute to this will arise from image acquisition settings. Improvements to summarization or targeting of vessel width and tortuosity measurements may enhance agreement and consequently increase the efficacy of these measures in clinical settings. A collaborative retinal imaging summit of clinicians and software developers would be of immense value in progressing standardization, identifying areas where there is a need for improvement, anticipating developments in imaging and measurement technology, developing recommendations, and facilitating consensus development of best practices. Future studies examining the impact of software-specific variability in relation to normative values and clinical cut-offs would further stimulate research into the retina as a source of reliable and accurate biomarkers.

## Supplementary Material

Supplement 1

Supplement 2

Supplement 3

## References

[1] Cheung CYL, Ong YT, Ikram MK, Chen C, Wong TY. Retinal microvasculature in Alzheimer's disease. *J Alzheimers Dis*. 2014; 42 suppl 4: S339– 352. 25351108 10.3233/JAD-141596

[2] Wong TY, Klein R, Klein BE, Tielsch JM, Hubbard L, Nieto FJ. Retinal microvascular abnormalities and their relationship with hypertension, cardiovascular disease, and mortality. *Surv Ophthalmol*. 2001; 46: 59– 80. 11525792 10.1016/s0039-6257(01)00234-x

[3] Taylor AM, MacGillivray TJ, Henderson RD, Retinal vascular fractal dimension, childhood IQ, and cognitive ability in old age: the Lothian birth cohort study 1936. *PLoS One*. 2015; 10: e0121119. 25816017 10.1371/journal.pone.0121119 PMC4376388

[4] McGrory S, Taylor AM, Kirin M, Retinal microvascular network geometry and cognitive abilities in community-dwelling older people: the Lothian Birth Cohort 1936 study. *Br J Ophthalmol*. 2016; 3090. 10.1136/bjophthalmol-2016-309017 PMC5530803 28400371

[5] Doubal FN, MacGillivray TJ, Hokke PE, Dhillon B, Dennis M S, Wardlaw J M. Differences in retinal vessels support a distinct vasculopathy causing lacunar stroke. *Neurology*. 2009; 72: 1773– 1778. 19451533 10.1212/WNL.0b013e3181a60a71 PMC2827311

[6] Perez-Rovira A, MacGillivray T, Trucco E, VAMPIRE: vessel assessment and measurement platform for images of the retina. *Conf Proc IEEE Eng Med Biol Soc*. 2011; 2011: 3391– 3394. 22255067 10.1109/IEMBS.2011.6090918

[7] Trucco E, Ballerini L, Relan D, Novel VAMPIRE algorithms for quantitative analysis of the retinal vasculature. *Proc Biosignals Biorobotics Conf*. 2013: 1– 4.

[8] Trucco E, Giachetti A, Ballerini L, Relan D, Cavinato A, MacGillivray T. Morphometric measurements of the retinal vasculature in fundus images with VAMPIRE. : Lim J-H, Ong S-H, Xiong W, *Biomedical Image Understanding*. Hoboken, NJ: John Wiley & Sons, Inc.; 2015: 91– 112.

[9] Fraz MM, Welikala RA, Rudnicka AR, Owen CG, Strachan DP, Barman SA. QUARTZ: Quantitative Analysis of Retinal Vessel Topology and size–an automated system for quantification of retinal vessels morphology. *Expert Sys*. 2015; 42: 7221– 7234.

[10] Bankhead P, Scholfield CN, McGeown JG, Curtis TM. Fast retinal vessel detection and measurement using wavelets and edge location refinement. *PLoS One*. 2012; 7: e32435. 22427837 10.1371/journal.pone.0032435 PMC3299657

[11] Couper DJ, Klein R, Hubbard LD, Reliability of retinal photography in the assessment of retinal microvascular characteristics: the Atherosclerosis Risk in Communities Study. *Am J Ophthalmol*. 2002; 133: 78– 88. 11755842 10.1016/s0002-9394(01)01315-0

[12] Ikram MK, Ong YT, Cheung CY, Wong TY. Retinal vascular caliber measurements: clinical significance, current knowledge and future perspectives. *Ophthalmologica*. 2013; 229: 125– 36. 23006932 10.1159/000342158

[13] Cosatto VF, Liew G, Rochtchina E, Retinal vascular fractal dimension measurement and its influence from imaging variation: results of two segmentation methods. *Curr Eye Res*. 2010; 35: 850– 856. 20795868 10.3109/02713683.2010.490628

[14] Huang F, Zhang J, Bekkers EJ, Dashtbozorg B, ter Haar Romeny BM. Stability analysis of fractal dimensions in retinal vasculature. : Liu J, Trucco E, Xu Y, Chen X, Garvin MK, *Proceedings of the Ophthalmic Medical Image Analysis Second International Workshop, OMIA 2015*, held in conjunction with MICCAI 2015, Lowa Research Online, Munich, Germany, 1– 8, 10 9, 2015.

[15] Huang F, Dashtbozorg B, Zhang J, Reliability of using retinal vascular fractal dimension as a biomarker in the diabetic retinopathy detection. *J Opthalmol*. 2016; 6259047. 10.1155/2016/6259047 PMC5040128 27703803

[16] Bussel II, Wollstein G, Schuman JS. OCT for glaucoma diagnosis, screening and detection of glaucoma progression. *Br J Ophthalmol*. 2014; 98 Suppl 2: ii15– ii19 24357497 10.1136/bjophthalmol-2013-304326 PMC4208340

[17] Hao H. *Analysis of Variation in Retinal Vascular Assessment*. Melbourne, Australia: RMIT University, 2014 Doctoral dissertation.

[18] Wei FF, Zhang ZY, Petit T, Retinal microvascular diameter, a hypertension-related trait, in ECG-gated vs. non-gated images analyzed by IVAN and SIVA. *Hypertens Res*. 2016; 39: 886– 892. 27383509 10.1038/hr.2016.81

[19] Yip W, Tham YC, Hsu W, Comparison of common retinal vessel caliber measurement software and a conversion algorithm. *Transl Vis Sci Technol*. 2016; 5: 11. 10.1167/tvst.5.5.11 PMC5063055 27752402

[20] Scottish Council for Research in Education. *The Intelligence of Scottish Children: A National Survey of an Age Group*. London, UK: University of London Press, Limited; 1933.

[21] Scottish Council for Research in Education. *The Trend of Scottish Intelligence: A Comparison of the 1947 and 1932 Surveys of the Intelligence of Eleven-Year-Old Pupils*. London, UK: University of London Press; 1949.

[22] Deary IJ, Whalley LJ, Starr JM. *The Scottish Mental Surveys of 1932 and 1947*. Washington, DC, US: American Psychological Association; 2009.

[23] Deary IJ, Gow AJ, Taylor MD, The Lothian Birth Cohort 1936: a study to examine influences on cognitive ageing from age 11 to age 70 and beyond. *BMC Geriatr*. 2007; 7: 28. 18053258 10.1186/1471-2318-7-28 PMC2222601

[24] Deary IJ, Gow AJ, Pattie A, Starr JM. Cohort profile: The Lothian Birth Cohorts of 1921 and 1936. *Int J Epidemiol*. 2012; 41: 1576– 1584. 22253310 10.1093/ije/dyr197

[25] Cheung CY, Tay WT, Mitchell P, Quantitative and qualitative retinal microvascular characteristics and blood pressure. *J Hypertens*. 2011; 2: 1380– 1391. 10.1097/HJH.0b013e328347266c 21558958

[26] Lau QP, Lee ML, Hsu W, Wong TY. The Singapore Eye Vessel Assessment System. : Ng EYK, Acharya UR, Campilo A, Suri JS, *Image Analysis and Modeling in Ophthalmology*. Boca Raton, FL: CRC Press; 2014: 143– 160.

[27] Giachetti A, Ballerini L, Trucco E. Accurate and reliable segmentation of the optic disc in digital fundus images. *J Med Imaging*. 2014; 1: 024001. 10.1117/1.JMI.1.2.024001 PMC4478982 26158034

[28] Relan D, MacGillivray T, Ballerini L, Trucco E. Automatic retinal vessel classification using a least square-support vector machine in VAMPIRE. *Conf Proc IEEE Eng Med Biol Soc*. 2014; 2014: 142– 145. 25569917 10.1109/EMBC.2014.6943549

[29] Lisowska A, Annunziata R, Trucco E, Karl D, Loh GK. An experimental assessment of five indices of retinal vessel tortuosity with the RET-TORT Public Dataset. *Conf Proc IEEE Eng Med Biol Sci*. 2014; 2014: 5414– 5417. 10.1109/EMBC.2014.6944850 25571218

[30] Pellegrini E, Robertson G, Trucco E. Blood vessel segmentation and width estimation in ultra-wide field scanning laser ophthalmoscopy. *Biomed Opt Express*. 2014; 5: 4329– 4337. 25574441 10.1364/BOE.5.004329 PMC4285608

[31] Jonas JB, Gusek GC, Naumann GO. Optic disc, cup and neuroretinal rim size, configuration and correlations in normal eyes. *Invest Ophthalmol Vis Sci*. 1988; 29: 1151– 1158. 3417404

[32] Sun C, Wang JJ, Mackey DA, Wong TY. Retinal vascular caliber: systemic, environmental and genetic associations. *Surv Ophthalmol*. 2009; 54: 74– 95. 19171211 10.1016/j.survophthal.2008.10.003

[33] Wardlaw JM, Allerhand M, Doubal FN, Vascular risk factors, large-artery atheroma, and brain white matter hyperintensities. *Neurology*. 2014; 82: 1331– 1338. 24623838 10.1212/WNL.0000000000000312 PMC4001185

[34] Lee J, Koh D, Ong CN. Statistical evaluation of agreement between two methods for measuring a quantitative variable. *Comput Biol Med*. 1989; 19: 61– 70. 2917462 10.1016/0010-4825(89)90036-x

[35] Bédard M, Martin NJ, Krueger P, Brazil K. Assessing reproducibility of data obtained with instruments based on continuous measurements. *Exp Aging Res*. 2000; 26: 353– 365. 11091941 10.1080/036107300750015741

[36] Portney LG, Watkins, MP. *Foundations of Clinical Research: Applications to Practice*. Upper Saddle River, NJ: Pearson/Prentice Hall; 2009.

[37] Bland JM, Altman DG. Comparing methods of measurement: why plotting difference against standard method is misleading. *Lancet*. 1995; 346: 1085– 1087. 7564793 10.1016/s0140-6736(95)91748-9

[38] Bland JM, Altman DG. Statistical methods for assessing agreement between two methods of clinical measurement. *Lancet*. 1986; 1: 307– 310. 2868172

[39] Benjamini Y, Hochberg Y. Controlling the false discovery rate: a practical and powerful approach to multiple testing. *J R Stat Soc Series B Stat Methodol*. 1995; 57: 289– 300.

[40] Williams EJ. The comparison of regression variables. *J R Stat Soc, Series B*. 1959; 21: 396– 399.

[41] Kottner J, Audigé L, Brorson S, Guidelines for reporting reliability and agreement studies (GRRAS) were proposed. *Int J Nurs Stud*. 2011; 48: 661– 671. 21514934 10.1016/j.ijnurstu.2011.01.016

[42] Heringa SM, Bouvy WH, Van Den Berg E, Moll AC, Kappelle LJ, Biessels GJ. Associations between retinal microvascular changes and dementia, cognitive functioning, and brain imaging abnormalities: a systematic review. *J Cereb Blood Flow Metab*. 2013; 33: 983– 995. 23591648 10.1038/jcbfm.2013.58 PMC3705441

[43] Soares JVB, Leandro JJG, Cesar RM, Jelinek HF, Cree MJ. Retinal vessel segmentation using the 2-D Gabor wavelet and supervised classification. *IEEE Trans Med Imaging*. 2006; 25: 1214– 1222. 16967806 10.1109/tmi.2006.879967

[44] Chen W, Sui L, Xu Z, Lang Y. Improved Zhang-Suen thinning algorithm in binary line drawing applications. International Conference on Systems and Informatics (ICSAI2012), Yantai, 2012; 1947– 1950. https://doi.org/10.1109/ICSAI.2012.6223430

[45] MacGillivray T, Patton N, Doubal FN, Graham C, Wardlaw JM. Fractal analysis of the retinal vascular network in fundus images. *Conf Proc IEEE Eng Med Biol Soc*. 2007: 6456– 6459. 18003503 10.1109/IEMBS.2007.4353837

[46] Lupaşcu CA, Tegolo D, Trucco E. Accurate estimation of retinal vessel width using bagged decision trees and an extended multiresolution Hermite model. *Med Image Anal*. 2013; 17: 1164– 1180. 24001930 10.1016/j.media.2013.07.006

[47] Al-Diri B, Hunter A, Steel D. An active contour model for segmenting and measuring retinal vessels. *IEEE Trans Med Imaging*. 2009; 28: 1488– 1497. 19336294 10.1109/TMI.2009.2017941

[48] Liew G, Sharrett AR, Kronmal R, Measurement of retinal vascular caliber: issues and alternatives to using the arteriole to venule ratio. *Invest Ophthalmol Vis Sci*. 2007; 48: 52– 57. 17197515 10.1167/iovs.06-0672

[49] Stosić T, Stosić BD. Multifractal analysis of human retinal vessels. *IEEE Trans Med Imaging*. 2006; 25: 1101– 1107. 16895002 10.1109/tmi.2006.879316

[50] Hoffmann EM, Zangwill LM, Crowston JG, Weinreb RN. Optic disk size and glaucoma. *Surv Ophthalmol*, 2007; 52: 32– 49. 17212989 10.1016/j.survophthal.2006.10.002 PMC1850981

[51] Biaggi P, Carasso S, Garceau P. Comparison of two different speckle tracking software systems: does the method matter? *Echocardiography*. 2011; 28: 539– 547. 21517954 10.1111/j.1540-8175.2011.01386.x

[52] Ather S, Iqbal F, Gulotta J. Comparison of three commercially available softwares for measuring left ventricular perfusion and function by gated SPECT myocardial perfusion imaging. *J Nucl Cardiol*. 2014; 21: 673– 681. 24715622 10.1007/s12350-014-9885-5

[53] Handayani A, Sijens PE, Lubbers DD, Triadyaksa P, Oudkerk M, van Ooijen PM. Influence of the choice of software package on the outcome of semiquantitative MR myocardial perfusion analysis. *Radiology*. 2013; 266: 759– 765. 23238157 10.1148/radiol.12120626

[54] Lujan BJ, Wang F, Gregori G, Calibration of fundus images using spectral domain optical coherence tomography. *Ophthalmic Surg Lasers Imaging Retina*. 2012; 39: S15– S20 10.3928/15428877-20080715-01 18777875

[55] Gavrielides MA, Zeng R, Kinnard LM, Myers KJ, Petrick N. Information-theoretic approach for analyzing bias and variance in lung nodule size estimation with CT: a phantom study. *IEEETrans Med Imaging*. 2010; 29: 1795– 1807. 10.1109/TMI.2010.2052466 20562039

[56] Peskin AP, Dima AA. Modeling clinical tumors to create reference data for tumor volume measurement. : Bebis G, Boyle R, Parvin B,, *Advances in Visual Computing*, Vol. 6454. Berlin, Heidelberg: Springer; 2010: 736– 746.

