# Supplementary material for: Towards Standardization of Quantitative Retinal Vascular Parameters: Comparison of SIVA and VAMPIRE Measurements in the Lothian Birth Cohort 1936
Source: Transl Vis Sci Technol. 2018 Mar 23;7(2):12. doi: 10.1167/tvst.7.2.12 (PMC5868859; doi:10.1167/tvst.7.2.12)
Supplement: Supplement 1 [file tvst-07-02-05_s01.pdf]

Supplementary Discussion

We identify seven potential sources of variation that will influence both software applications and may give rise to systematic differences between them. While not all factors are relevant to the results of the current study we include them here as potential causes of significant variations in measurements, hence requiring consideration in the interest of progressing the field towards standardization.

Imaging instruments. During image acquisition, the use of different imaging instruments will likely influence outcomes due to instrument specific factors, including the size of field of view and magnification. [1, 2]

Retinal field. The choice of imaging field may also affect summative outcomes: if the imaging captures different regions of the retina, different vessels or segments of vessels are observed, which may result in non-comparable summative measurements.

Image resolution. Differences in image resolution (microns per pixel), resulting from the sensor used to capture and generate the image, can also have a considerable effect on algorithm performance. [1]

(Note these factors are not an issue in the current study as camera system, imaging field and resolution was consistent across images).

Inter/intra-operator reliability. A recent analysis of inter-operator variability in VAMPIRE found good agreement between operators for measurements of branching coefficients (ICC>0.80) with lower levels of agreement for tortuosity (ICC <0.45) [3] In a similar, independent analysis, SIVA retinal width, fractal and tortuosity measurements demonstrated good inter-operator reliability (ICC>0.88).[4] It was not possible to assess intra-operator reliability in the current study due to data limitations, however this is a potential source of uncertainty.

Manual interaction. The differing degrees of interaction available to the user in different software applications is also likely to result in measurement variability. The amount of manual input in both software applications could be a potentially major source of bias when combined with other sources of variation.

Pulse cycle variations. Several studies have examined vessel width variations in pulse cycle. [5-9] While some results appear to be conflicting, Hao et al. [6] reported a mean variation of less than 4 microns in vessel width, but this is lower than the resolution of a typical fundus camera system and suggests any potential change in vessel width would be difficult to detect without appropriate instruments.

Physiological characteristics. Further variability may also be introduced from characteristics of the eye as an optical system, e.g. refractive error, axial length, lens opacity etc. Axial length was not measured in the Lothian Birth Cohort. Accurate and meaningful interpretation of retinal measurements requires not only precise quantification and calculation but also an understanding of potential effects of axial length and ocular refractive error. Future studies should compare metrics based on axial length as a source of retinal magnification conversion.

References

1. Trucco E, Ballerini L, Relan D, et al. Novel VAMPIRE algorithms for quantitative analysis of the retinal vasculature. *Proc. Biosignals Biorobotics Conf*. 2013:1-4.

2. Patton N, Aslam TM, MacGillivray T. et al. Retinal image analysis: concepts, applications and potential. *Prog Retin Res.* 2006;25(1):99-127

3. MacGillivray TJ, Cameron JR., Zhang Q. et al. Suitability of UK Biobank retinal images for automatic analysis of morphometric properties of the vasculature. *PloS one*. 2016;10(5):e0127914.

4. Kirin M, Nagy R, MacGillivray, TJ. et al. Determinants of retinal microvascular features and their relationships in two European populations. *J Hypertens.* 2017; *35*(8): 1646.

5. Knudtson MD, Lee KE, Hubbard LD, Wong TY, Klein R, Klein BE. Revised formulas for summarizing retinal vessel diameters. *Curr Eye Res.* 2003;27(3):143-149

6. Hao HAO, Sasongko MB, Wong TY. Does Retinal Vascular Geometry Vary with Cardiac Cycle? Retinal Vessel Geometry during Cardiac Cycle. *Invest Ophthalmol Vis Sci*. 2012; 53(9): 5799-5805.

7. Kumar DK, Aliahmad B, Hao H, Che Azemin MZ, Kawasaki R. A Method for Visualization of Fine Retinal Vascular Pulsation Using Nonmydriatic Fundus Camera Synchronized with Electrocardiogram*. ISRN Ophthalmology*. 2013;2013:865834. doi:10.1155/2013/865834.

8. Yilmaz I, Perente I, Kesim C, Saracoglu B, Yazici AT, Taskapili, M. Ocular pulse amplitude and retinal vessel caliber changes after intravitreal dexamethasone implant. *Middle East Afr J Ophthalmol*, 2016;23(3):250.

Supplementary Table S1. SIVA and VAMPIRE operator interaction

	SIVA	VAMPIRE
Main interactive components	Manually edit inaccurate optic disc detection<br>Options to open, save, undo, redo and select various image processing and editing methods<br>Visualization of the extracted vessel centerlines allowing the user to correct the lines and reclassify erroneously identified vessels as arteries or veins<br>Visualization of a selected line segment to allow the operator to delete unreliable samples and adjust the sampled widths<br>Display of a list of measurement output. Vessels with suspiciously high standard deviations of width are highlighted in red for the operator's attention	Manually edit inaccurate optic disc/fovea detection<br>Options to open, save, undo, redo<br>Visualization of the identified vessels allowing the user to reclassify erroneously identified vessels as arteries and veins<br>Visualization of vessel width boundaries to allow the operator to delete inaccurate vessel segments
Average manual image processing time per image	15 minutes (Ng et al. 2014)	4 minutes (MacGillivray et al. 2015)

References

MacGillivray TJ, Cameron JR, Zhang Q, El-Medany A, Mulholland C, Sheng Z, .. & Sudlow C. Suitability of UK Biobank retinal images for automatic analysis of morphometric properties of the vasculature. *PloS one.* 2015; 10(5): e0127914.)

Ng EY, Acharya UR, Suri JS, Campilho A. (Eds.). Image Analysis and Modeling in Ophthalmology: Boca Raton, FL, US: CRC Press; 2014: 143-160.

Supplementary Table S2. Description of retinal parameters with relevant software specific retinal zone and reference

Parameter	Description	Retinal Zone	Reference	
			SIVA	VAMPIRE
CRAE	Central Retinal Artery Equivalent	B	Hubbard et al. 1999	Patton, IOVS 2006
CRVE	Central Retinal Vein Equivalent	B	Knudston et al. 2003	
AVR	CRAE/CRVE=AVR	B		
FDa	Fractal dimension of arteriolar network	C	Manister, 1990 Liew et al. 2008	Stosic, TMI, 2006
FDv	Fractal dimension of venular network	C		
TORTa	Tortuosity arteriole	C	Sasongko et al. 2010. Cheung et al. 2011	Annunziata et al., MICCAI – OMIA, 2014
TORTv	Tortuosity venule	C		

References

Knudtson MD, Lee KE, Hubbard LD, Wong TY, Klein R, Klein BE. Revised formulas for summarizing retinal vessel diameters. Curr Eye Res. 2003; 27(3):143-149.

Hubbard LD, Brothers RJ, King WN. Methods for evaluation of retinal microvascular abnormalities associated with hypertension/sclerosis in the Atherosclerosis Risk in Communities Study. *Ophthalmology*. 1999; 106(12): 2269-2280.

Patton N, Aslam T, MacGillivray T, Dhillon B, Constable I. Asymmetry of retinal arteriolar branch widths at junctions affects ability of formulae to predict trunk arteriolar widths. *Invest Ophthalmol Vis Sci.* 2006;47(4):1329-1333.

Mainster MA. The fractal properties of retinal vessels: embryological and clinical implications. *Eye*. 1990;4(1):235-241.

Liew G, Wang JJ, Cheung N. The retinal vasculature as a fractal: methodology, reliability, and relationship to blood pressure. *Ophthalmology*. 2008;115(11): 1951-1956.

Stosic T, Stosic BD. Multifractal analysis of human retinal vessels. *IEEE Trans Med Imaging*. 2006;25(8):1101-1107.

Sasongko MB, Wang JJ, Donaghue KC. Alterations in retinal microvascular geometry in young type 1 diabetes. *Diabetes Care*. 2010; 33(6):1331-1336.

Cheung CYL, Zheng Y, Hsu W. Retinal vascular tortuosity, blood pressure, and cardiovascular risk factors. *Ophthalmology*, 2011;118(5);812-818.

Annunziata R, Kheirkhah A, Hamrah P, Trucco E. (2015, October). Scale and curvature invariant ridge detector for tortuous and fragmented structures. In International Conference on Medical Image Computing and Computer-Assisted Intervention (pp. 588-595). Springer International Publishing.

Supplementary Table S3. Difference in VAMPIRE CRAE and CRVE measurements based on individual and average image conversion factor in small subsample of LBC1936

	Pixel			ICF		CRAE micron			CRVE micron		
	OD diameter	CRAE	CRVE	Individual ICF formula	ICF	Individual ICF	Average ICF	Difference (Individual-Average)	Individual ICF	Average ICF	Difference (Individual-Average)
1st quintile	283.3	27.0	29.3	1800/283.2	6.35	171.4	113.3	58.17	186.0	131.7	54.32
	301.8	23.5	43.2	1800/301.7	5.96	140.0	136.3	3.66	257.3	181.1	76.15
2nd quintile	395.2	32.0	44.3	1800/395.2	4.55	145.4	134.1	11.29	201.7	186.0	15.67
	395.3	32.1	45.0	1800/395.3	4.55	145.9	134.6	11.34	204.6	188.7	15.92
3rd quintile	425.2	38.0	49.0	1800/425.2	4.23	161.1	159.5	1.57	207.1	205.5	1.68
	425.5	28.4	43.7	1800/425.5	4.23	120.0	119.0	0.97	184.8	183.3	1.49
4th quintile	458.1	31.6	45.8	1800/458.1	3.93	124.1	132.5	-8.39	180.0	192.2	-12.20
	458.7	34.8	39.2	1800/458.7	3.92	136.5	146.1	-9.62	153.8	164.7	-10.83
5th quintile	644.0	33.4	47.8	1800/644.0	2.79	93.2	140.1	-46.96	133.5	200.8	-67.28
	650.9	31.0	44.5	1800/650.9	2.76	85.7	130.2	-44.67	122.8	186.7	-63.88

Note. OD=optic disc; CRAE=central retinal artery equivalent; CRVE=central retinal vein equivalent; ICF=image conversion factor; individual ICF=image conversion factor based on participants optic disc diameter; average ICF=image conversion factor based on average optic disc diameter of sample

Supplementary Table S4. Difference between mean VAMPIRE CRAE and CRVE micron measurements based on mean OD diameter image conversion and participant specific OD diameter image conversion

	Mean (SD)			Mean (SD)		
	Individual ICF	Average ICF	*p*	Individual ICF	Average ICF	*p*
Table 1 sample (n=10)	132.3 (27.3)	134.6 (13.0)	0.816	183.2 (39.3)	182.1 (20.9)	0.940
Whole sample (n=665)	133.2 (18.3)	131.6 (10.8)	0.004*	180.9 (24.4)	178.9 (15.7)	0.007*

Note. ICF=image conversion factor; SD=standard deviation
*p<0.05
