# Supplementary material for: Towards Standardization of Quantitative Retinal Vascular Parameters: Comparison of SIVA and VAMPIRE Measurements in the Lothian Birth Cohort 1936
Source: Transl Vis Sci Technol. 2018 Mar 23;7(2):12. doi: 10.1167/tvst.7.2.12 (PMC5868859; doi:10.1167/tvst.7.2.12)
Supplement: Supplement 2 [file tvst-07-02-05_sf01.pdf]

Supplementary Figure S1.

SIVA and VAMPIRE interface

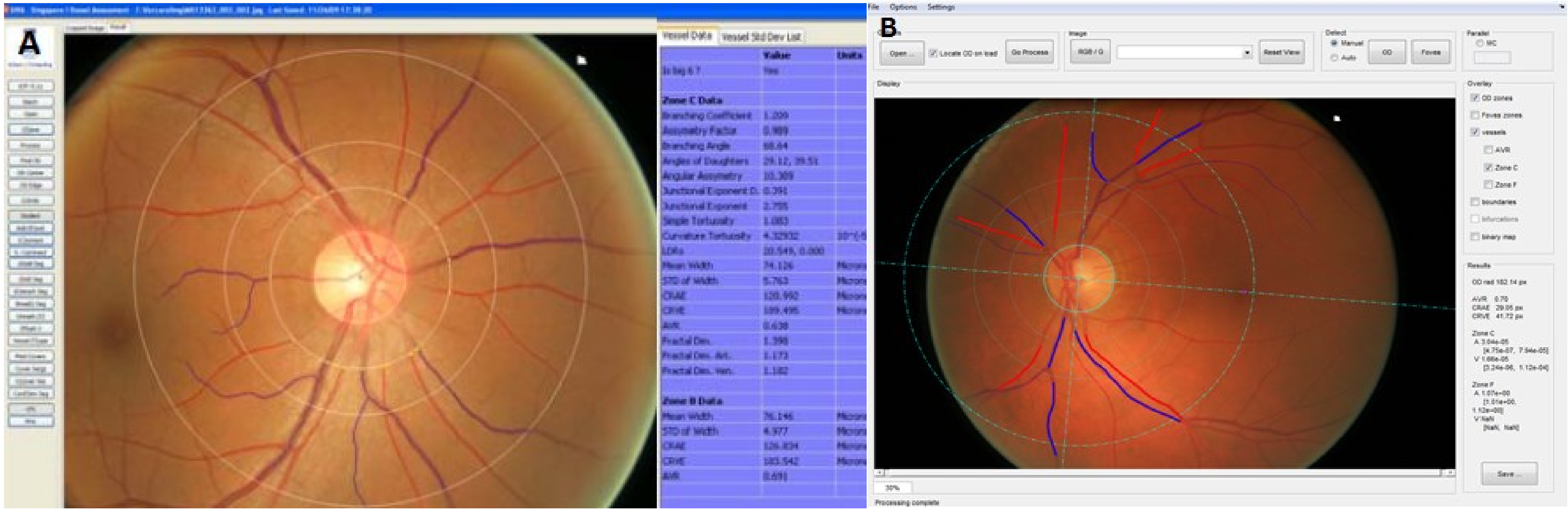

Note. A = SIVA interface; B = VAMPIRE interface.

SIVA image: Singaporeeyeresearchinstitute. 2011. Singapore “I” Vessel Assessment (SIVA): Computer-aided Integrated Platform for Large-scale Non-invasive Observation of Cardiovascular Disorders Using Retina Image Analysis [Online]. Available: https://www.etpl.sg/qql/slot/u94/Software%20to%20License/SIVA/SIVA%20eb rochure.pdf 2016]
