# Supplementary material for: Towards Standardization of Quantitative Retinal Vascular Parameters: Comparison of SIVA and VAMPIRE Measurements in the Lothian Birth Cohort 1936
Source: Transl Vis Sci Technol. 2018 Mar 23;7(2):12. doi: 10.1167/tvst.7.2.12 (PMC5868859; doi:10.1167/tvst.7.2.12)
Supplement: Supplement 3 [file tvst-07-02-05_sf02.pdf]

Supplementary Figure S2.

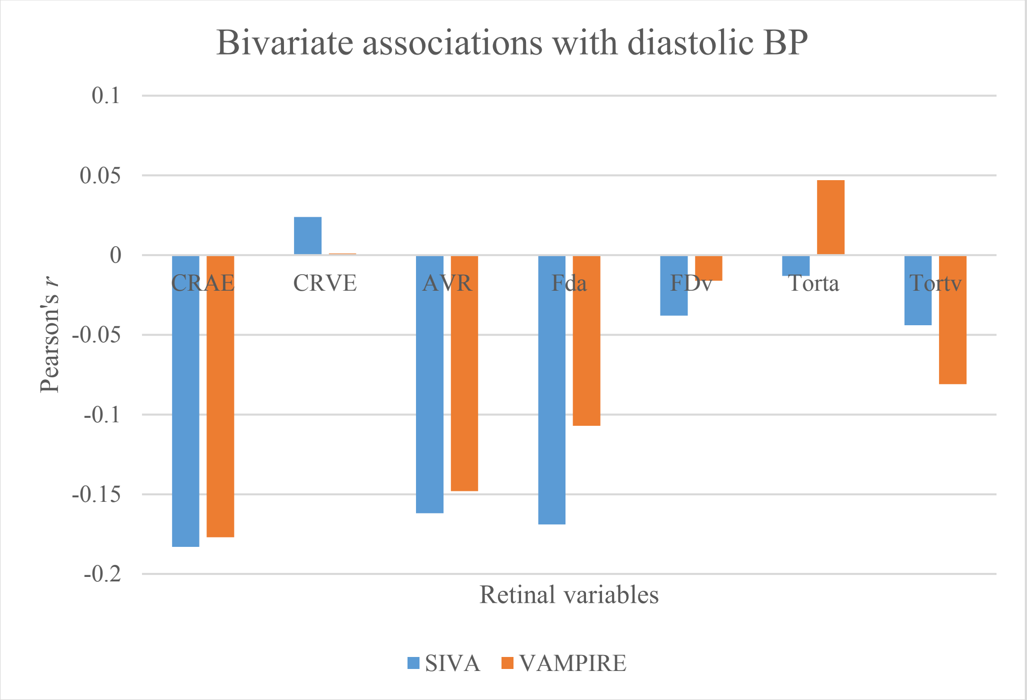

Supplementary Figure S1 illustrating intersoftware agreement in retinal-diastolic blood pressure correlation magnitudes.

Note. Associations significant at absolute *r* > .078
